# Epidemiological patterns and antimicrobial resistance of bacterial diarrhea among children in Nairobi City, Kenya 

**Published:** 2020

**Authors:** Mark Kilongosi Webale, Christine Wanjala, Bernard Guyah, Nathan Shaviya, Godwil O. Munyekenye, Peter Lokamar Nyanga, Immaculate Nyaseba Marwa, Sammy Kagoiyo, Laura Nyawira Wangai, Sella K. Webale, Kenny Kamau, Nicholas Kitungulu

**Affiliations:** 1 *School of Health Sciences, Kirinyaga University, Kutus, Kenya*; 2 *School of Public Health, Biomedical Sciences and Technology, Masinde Muliro University of Science and Technology, Kakamega, Kenya*; 3 *School of Public Health, Biomedical Sciences and Technology, Maseno University, Maseno, Kenya*; 4 *Disease Surveillance and Response Unit, Ministry of Health, Nairobi City, Kenya*; 5 *School of Biological Sciences, Masinde Muliro University of Science and Technology, Kakamega, Kenya*

**Keywords:** Epidemiology, antimicrobial resistance, bacterial diarrhea

## Abstract

**Aim:**

Determine the prevalence of enteric bacterial pathogens and their antimicrobial resistance among diarrheic children in Nairobi City, Kenya.

**Background:**

Regardless of enteric bacterial pathogens being a major cause of gastroenteritis in children, their occurrence and antimicrobial resistance patterns reveals regional spatial and temporal variation.

**Methods:**

In a cross-sectional study, a total of 374 children below five years presenting with diarrhea at Mbagathi County Hospital were recruited. Stool microbiology test was used to detect enteric bacterial infection. Antimicrobial resistance was determined using the disk diffusion method.

**Results:**

Diarrheagenic *E. coli* (36.4%) was the leading species followed by *Shigella* (3.2%), *Salmonella* (2.4%), *Campylobacter* (1.6%), *Yersinia* (1.3%) and *Aeromonas* (1.1%) species. *Escherichia coli* pathotyping revealed that 20.9%, 4.0%, 10.2% and 0.5% of the study participants were infected with enteroaggregative *E. coli* (EAEC), enteropathogenic *E. coli* (EPEC), enterotoxigenic *E. coli* (ETEC) and enteroinvasive *E. coli* (EIEC) pure isolates while the prevalence of mixed pathotype infections was 0.3% for EAEC/EPEC/ETEC and 0.5% for EAEC/ETEC. *Shigella* sero-grouping revealed that 0.5%, 0.3%, 1.9%, and 0.5% were infected with *Shigella boydii*, *Shigella dysentriae*, *Shigella flexneri* and *Shigella sonnei* pure isolates. *Shigella *species and *E. coli* co-infection was detected in 2.4% of the children, specifically, 1.1% for EAEC/*Shigella boydii*, 0.5% for EAEC/*Shigella dysentriae* and 0.3% in each case of EAEC/*Shigella sonnei*, EPEC/*Shigella flexneri* and ETEC/*Shigella flexneri* co-infections. Most of the isolates were resistant to commonly prescribed antibiotics.

**Conclusion:**

There was a high prevalence of enteric bacterial pathogens and co-infection alters epidemiological dynamics of bacterial diarrhea in children. Continuous antibiotic resistance surveillance is justified because the pathogens were highly resistant to commonly prescribed antimicrobials.

## Introduction

 In the year 2016, diarrhea accounted for more than 1.6 million deaths globally making it the fifth leading cause of mortality among children less than 5 years (1). The majority of these deaths occurred in resource-limited continents including Africa (1, 2). In Sub-Saharan Africa, there were more than 1.2 billion cases of diarrhea, of which 371 million, leading to at least 0.2 million deaths, occurred in children younger than five years (1, 2). Besides parasites and viruses, enteric bacteria continue to be a leading cause of gastroenteritis in children (1). 

Enteric bacterial pathogens accounts for more than 300 million episodes of diarrhea and one million deaths among children globally, the majority of which are caused by *Escherichia coli* and the remainder being *Campylobacter, Shigella*, *Vibrio* and *Salmonella species *(1). Previous studies across Kenya demonstrated that *Shigella*, *E. coli*, and *Salmonella* species were associated with childhood diarrhea (3), while others did not (4), suggesting geographic uniqueness of gut microbiota in immunity towards enteric infection patterns in Kenya (5-8). Also, previous studies across Kenya found an association between *Campylobacter* and *Shigella* species with diarrhea among HIV infected children (9) while others did not (10), signifying that HIV alters the already geographic unique gut microbiota profile which is critical for host immunity to enteric bacterial infections (11, 12). The risk factors of enteric bacterial pathogens, however, appear to be distributed differently (3, 13, 14) and as a result, the incidences of specific pathogens differ across Kenya (4, 14-20). A more recent study in Nairobi city identified *Yersinia enterocolitica* as an etiology of childhood diarrhea (3) while previous studies did not (13, 17), indicating the emergence and spread of new pathogen strains driven by poor water, sanitation and hygiene (WASH) practices in the city (3) qualifying the need for constant epidemiological surveillance in Nairobi city, Kenya. However, there is paucity of information on the prevalence of enteric bacterial pathogens among diarrheic children in Nairobi city, Kenya. 

The innovation of antibiotics led to optimism that enteric bacterial infections could be controlled and prevented. However, enteric bacteria resistance to antibiotics is still the leading cause of death globally, including Kenya (21). Many studies have reported that enteropathogenic bacteria isolated from diarrheic children in Kenya including Nairobi city, can develop resistance (3, 13, 14, 22, 23). For instance, a previous study in Nairobi city reported the resistance of *E. coli* and *Shigella* isolates to ampicillin, trimethoprim/sulfamethoxazole, streptomycin, chloramphenicol and tetracycline (3) while a recent study showed changing patterns with increasing resistance (23). This increasing resistance is due to inappropriate antibiotic use increasing selection and transmission of antibiotic resistant strains in the city (3, 13, 22, 24, 25) in this era when there is a serious lack of new antibiotics under development to combat the growing antimicrobial resistance (26, 27), justifying the need for continuous antimicrobial resistance surveillance. However, there is limited information on the prevalence of antimicrobial resistance of enteric bacterial pathogens among children in Nairobi city, Kenya. This study, therefore, aimed at determining the prevalence of enteric bacterial pathogens and their antimicrobial resistance among diarrheic children in Nairobi city, Kenya. 

## Methods

**Study site, design and population**

A detailed description of the study site, design and population is presented here (23). Briefly, this was a cross-sectional study targeting diarrheic children <5 years, seeking treatment at Mbagathi hospital, Nairobi city, Kenya. Diarrhea was defined according to World Health Organization (WHO) guidelines as the occurrence of three or more loose, liquid, or watery stools in a 24-hour period (28). Demographic and clinical information of the study participants were collected using a questionnaire. Stool specimens were collected and microbiology laboratory analysis performed within two hours of collection. Stool samples of children who had received antibiotics and who did not provide informed consent were excluded from the study.

**Bacteriological procedures**

Identification of bacteria species was performed according to WHO recommendations (29). All fecal specimens were cultured in alkaline peptone water, selenite broth and blood agar enrichment media, followed by sub-culture on selective and differential media as described elsewhere (18). Isolates were then subjected to Gram staining and biochemical tests using oxidase test, lysine decarboxylase test, urease test, citrate test, hydrogen sulfide gas production, fermentation test, and motility test to identify the significant characteristic of bacteria according to the standard methods (29). *E. coli* pathotyping and *Shigella* sero-grouping was performed as previously described (23).

**Antimicrobial Resistance**

Antibiotic susceptibility was performed using Kirby-Bauer disk diffusion method on Mueller Hinton agar by incubating at 37°C for 18 hours (30). Broth turbidity was made to match with 0.5 McFarland standards. Antibiotics discs of ampicillin, trimethoprim/sulfamethoxazole, ceftriaxone, streptomycin, amoxicillin/clavulanic acid, gentamycin, kanamycin, ciprofloxacin, chloramphenicol, erythromycin, nalidixic acid and tetracycline were tested.

**Data analysis**

Statistical analyses were performed using SPSS version 19.0 for Windows (IBM SPSS Statistics for Windows, Version 19.0. Armonk, NY: IBM Corp.). Descriptive statistics, namely frequencies and percentages, were used to present demographic and clinical data, frequency of enteric bacteria pathogens and their antimicrobial susceptibility pattern. 

**Ethical considerations**

This study was ethically approved by Kenyatta National Hospital/University of Nairobi (KNH-UoN) Ethics and Research Committee and was conducted according to the Declaration of Helsinki (31). A consent form was read and signed by either parent or guardian of each child. Diarrheic children were treated by clinicians according to World Health Organization (WHO) guidelines for treatment of diarrhea in children (28). All study participants’ information and test results were kept confidential. The results of bacterial cultures were used in clinical management of study participants. 

## Results

**Demographic and clinical information of study participants**

The demographic and clinical information of study participants is presented in table 1. A total of 374 children were recruited in the study. Age distribution showed that, out of the total study subjects, 242 (70.0%) were between 1 and 36 months and 112 (30.0%) children were between 37 and 60 months. The overall gender distribution was 181 (48%) females and 193 (52%) males. Guardians of 371 (99.2%) and 3 (0.8%) reported using piped and borehole water, respectively. In addition, 220 (58.8%) reported treating drinking water. Occupation distribution showed that 2 (0.5%), 17 (4.5%), 5 (1.3%), 14 (3.7%), and 178 (47.6%) of the guardians were employed in the fields of healthcare, office administrative support, construction/installation/repair, education/ training, and sales, respectively, while 158 (42.2%) were unemployed. 

**Table 1:** Demographic and clinical information of study participants

Characteristics	Number (%)
Age in months	
1-36	242 (70.0)
37-60	112 (30.0)
Gender	
Female	181 (48)
Male	193 (52)
Source of water	
Piped water	371 (99.2)
Borehole	3 (0.8)
Water treatment	220 (58.8)
Occupation of guardian	
Health care practitioner	2 (0.5)
Office/administrative/support	17 (4.5)
Construction/installation/repair	5 (1.3)
Education/ training	14 (3.7)
Sales	178 (47.6)
Unemployed	158 (42.2)
Body temperature	
<38.0	58 (15.5)
≥ 38.0	316 (84.5)
Duration of diarrhea	
1-3	308 (82.4)
4-6	35 (9.4)
≥7	31 (8.3)
Symptoms	
Vomiting	298 (79.7)
Fever	310 (82.9)
Abdominal cramp	251 (67.1)
Headache	12 (3.2)
Nausea	50 (13.4)
Appetite loss	345 (92.2)
Sunken eyeballs	311 (83.2)
Dry tongue	117 (31.3)
Reduced skin elasticity	192 (51.3)

Data are presented as number and proportions (%) of study participants. ≤, less than or equal to. <, less than. ≥, greater than or equal to. >, greater than.

Temperature of <38.0°C and ≥ 38.0°C was recorded in 58 (15.5%) and 316 (84.5%) children, respectively. In this study, 308 (82.4%), 35 (9.4%) and 31 (8.3%), respectively, reported having diarrhea for 1-3, 4-6 and ≥7 days. Vomiting was reported in 298 (79.7%) patients, fever in 310 (82.9%), abdominal cramp in 251 (67.1%), headache in 12 (3.2%), nausea in 50 (13.4%), and appetite loss in 345 (92.2%) children. Clinical diagnosis of dehydration revealed that 311 (83.2%) had sunken eyeballs, 117 (31.3%) children had dry tongues and 192 (51.3%) had reduced skin elasticity.

**Table 2:** Prevalence of enteric bacterial pathogens isolated from study participants

Enteropathogenic bacteria	N (%)
Diarrheagenic *E. coli*	136 (36.4)
EAEC	78 (20.9)
EPEC	15 (4.0)
ETEC	38 (10.2)
EIEC	2 (0.5)
EAEC/EPEC/ETEC	1 (0.3)
EAEC/ETEC	2 (0.5)
*Salmonella species*	9 (2.4)
*Shigella species*	12 (3.2)
* Shigella boydii*	2 (0.5)
* Shigella dysentriae*	1 (0.3)
* Shigella flexneri*	7 (1.9)
* Shigella sonnei*	2 (0.5)
*Campylobacter* species	6 (1.6)
*Yersinia* *enterocolitica*	5 (1.3)
*Aeromonas* species	4 (1.1)
*Shigella *species*/E. coli* co-infection	9 (2.4)
EAEC*/Shigella boydii*	4 (1.1)
EAEC*/Shigella dysentriae*	2 (0.5)
EAEC*/Shigella sonnei*	1 (0.3)
EPEC*/Shigella flexneri*	1 (0.3)
ETEC*/Shigella flexneri*	1 (0.3)

Data are presented as number and proportions (%) of study participants. *E. coli*, *Escherichia coli*. EPEC, enteropathogenic *E. coli*. ETEC, enterotoxigenic *E. coli*. EAEC, enteroaggregative *E. coli*. EIEC, enteroinvasive *E. coli*.

**Prevalence of enteric bacteria pathogens isolated from study participants**

The prevalence of enteric bacterial pathogens is presented in table 2. A total of 136 (36.4%) children were infected by diarrheagenic *E. coli*. Of these, 78 (20.9%), 15 (4.0%), 38 (10.2%) and 2 (0.5%) children were infected with enteroaggregative *E. coli* (EAEC), enteropathogenic *E. coli* (EPEC), enterotoxigenic *E. coli* (ETEC) and enteroinvasive *E. coli* (EIEC) pure strains, respectively, while mixed pathotype infections was detected in 1 (0.3%) child for EAEC/EPEC/ETEC and 2 (0.5%) children for EAEC/ETEC. *Salmonella* species was detected in stool samples of 9 (2.4%) children. There were 12 (3.2%) children infected with *Shigella *species*, *of which 2 (0.5%), 1 (0.3%), 7 (1.9%), and 2 (0.5%) were infected with *Shigella boydii, Shigella dysentriae, Shigella flexneri *and* Shigella sonnei, *respectively*.* *Campylobacter*, *Yersinia* and *Aeromonas* species were detected in stool samples of 6 (1.6%), 5 (1.3%) and 4 (1.1%) children, respectively. There were 9 (2.4%) children co-infected with *Shigella *species and *E. coli, *of which, 4 (1.1%) were EAEC*/Shigella boydii *co-infections*, *2 (0.5%) were EAEC*/Shigella dysentriae* co-infections, while one (0.3%) case was reported for EAEC/*Shigella sonnei*, EPEC/*Shigella flexneri* and ETEC/*Shigella flexneri* co-infection.

**Antimicrobial susceptibility patterns of enteric bacterial pathogens isolated from study participants**

The antimicrobial susceptibility patterns of the enteric bacterial pathogens isolated from diarrheic children in Nairobi city, Kenya, are presented in table 3. About 80 (55.2%), 89 (61.4%), 18 (12.4%), 91 (76.6%), 38 (26.2%), 69 (47.6%), 91 (62.8%), 41 (28.3%), 90 (62.1%), 12 (8.3%), 16 (11.0%), and 120 (82.8%) of diarrheagenic *E. coli *were resistant to ampicillin, trimethoprim/sulfamethoxazole, ceftriaxone, streptomycin, amoxicillin/clavulanic acid, gentamycin, kanamycin, ciprofloxacin, chloramphenicol, erythromycin, nalidixic acid and tetracycline, respectively. Although none of the *Salmonella* isolate was resistant to trimethoprim/sulfamethoxazole, gentamycin and nalidixic acid, 7 (77.8%), 7 (77.8%), 9 (100.0%), 5 (55.6%), 8 (88.9%), 8 (88.9%), 7 (77.8%), and 6 (66.7%) of *Salmonella* species isolates were resistant to ampicillin, ceftriaxone, streptomycin, amoxicillin/clavulanic acid, kanamycin, ciprofloxacin, chloramphenicol, erythromycin and tetracycline, respectively. 

*Shigella* species resistant to kanamycin was not detected, nevertheless, 13 (28.6%), 10 (47.6%), 10 (47.6%), 12 (57.1%), 4 (19.0%), 2 (9.5%), 4 (19.0%), 12 (57.1%), 2 (9.5%), 4 (19.0%) and 14 (66.7%) of the *Shigella* isolates were resistant to ampicillin, trimethoprim/sulfamethoxazole, ceftriaxone, streptomycin, amoxicillin/clavulanic acid, gentamycin, ciprofloxacin, chloramphenicol, erythromycin, nalidixic acid and tetracycline, respectively. While *Campylobacter* species was not resistant to streptomycin, amoxicillin/clavulanic acid, kanamycin, ciprofloxacin and nalidixic acid, 3 (50.0%), 1 (16.7%), 1 (16.7%), 1 (16.7%), 4 (66.7%), 3 (50.0%), and 5 (83.3%) of the *Campylobacter *isolates were resistant to ampicillin, trimethoprim/sulfamethoxazole, ceftriaxone, gentamycin, chloramphenicol, erythromycin, tetracycline, respectively. *Yersinia *was not resistant to trimethoprim/sulfamethoxazole, ciprofloxacin, and nalidixic acid. 

**Table 3:** Antimicrobial susceptibility patterns of enteric bacterial pathogens isolated from study participants

Enterpathogenic bacteria	Antibiotic	Susceptibility pattern (%)		
		Sensitive	Intermediate	Resistant
Diarrheagenic *E. coli*	Ampicillin	22 (15.2)	43 (29.7)	80 (55.2)
	Trimethoprim/sulfamethoxazole	25 (17.2)	31 (21.4)	89 (61.4)
	Ceftriaxone	106 (73.1)	21 (14.5)	18 (12.4)
	Streptomycin	34 (23.4)	20 (13.8)	91 (76.6)
	Amoxicillin/clavulanic acid	87 (60.0)	20 (13.8)	38 (26.2)
	Gentamycin	48 (33.1)	28 (19.3)	69 (47.6)
	Kanamycin	10 (6.9)	44 (30.3)	91 (62.8)
	Ciprofloxacin	94 (64.8)	10 (6.9)	41 (28.3)
	Chloramphenicol	14 (9.7)	41 (28.3)	90 (62.1)
	Erythromycin	81 (55.9)	52 (35.9)	12 (8.3)
	Nalidixic acid	99 (68.3)	30 (20.7)	16 (11.0)
	Tetracycline	15 (10.3)	10 (6.9)	120 (82.8)
*Salmonella* species	Ampicillin	0 (0.0)	2 (22.2)	7 (77.8)
	Trimethoprim/sulfamethoxazole	9 (100.0)	0 (0.0)	0 (0.0)
	Ceftriaxone	2 (22.2)	0 (0.0)	7 (77.8)
	Streptomycin	0 (0.0)	0 (0.0)	9 (100.0)
	Amoxicillin/clavulanic acid	4 (44.4)	0 (0.0)	5 (55.6)
	Gentamycin	9 (100.0)	0 (0.0)	0 (0.0)
	Kanamycin	1 (11.1)	0 (0.0)	8 (88.9)
	Ciprofloxacin	0 (0.0)	1 (11.1)	8 (88.9)
	Chloramphenicol	2 (22.2)	0 (0.0)	7 (77.8)
	Erythromycin	1 (11.1)	2 (22.2)	6 (66.7)
	Nalidixic acid	9 (100.0)	0 (0.0)	0 (0.0)
	Tetracycline	3 (33.3)	1 (11.1)	5 (55.6)
*Shigella* species	Ampicillin	2 (9.5)	6 (28.6)	13 (28.6)
	Trimethoprim/sulfamethoxazole	8 (38.1)	3 (14.3)	10 (47.6)
	Ceftriaxone	11 (52.4)	0 (0.0)	10 (47.6)
	Streptomycin	2 (9.5)	7 (33.3)	12 (57.1)
	Amoxicillin/clavulanic acid	16 (76.2)	1 (4.8)	4 (19.0)
	Gentamycin	18 (85.7)	1 (4.8)	2 (9.5)
	Kanamycin	4 (19.0)	17 (81.0)	0 (0.0)
	Ciprofloxacin	11 (52.4)	6 (28.6)	4 (19.0)
	Chloramphenicol	5 (23.8)	4 (19.0)	12 (57.1)
	Erythromycin	5 (23.8)	14 (66.7)	2 (9.5)
	Nalidixic acid	13 (62.0)	4 (19.0)	4 (19.0)
	Tetracycline	4 (19.0)	3 (14.3)	14 (66.7)
*Campylobacter *species	Ampicillin	2 (33.3)	1 (16.7)	3 (50.0)
	Trimethoprim/sulfamethoxazole	5 (83.3)	0 (0.0)	1 (16.7)
	Ceftriaxone	4 (66.7)	1 (16.7)	1 (16.7)
	Streptomycin	5 (83.3)	1 (16.7)	0 (0.0)
	Amoxicillin/clavulanic acid	6 (100.0)	0 (0.0)	0 (0.0)
	Gentamycin	5 (83.3)	0 (0.0)	1 (16.7)
	Kanamycin	4 (66.7)	2 (33.3)	0 (0.0)
	Ciprofloxacin	6 (100.0)	0 (0.0)	0 (0.0)
	Chloramphenicol	0 (0.0)	1 (33.3)	4 (66.7)
	Erythromycin	1 (16.7)	2 (33.3)	3 (50.0)
	Nalidixic acid	4 (66.7)	2 (33.3)	0 (0.0)
	Tetracycline	1 (16.7)	0 (0.0)	5 (83.3)
*Yersinia enterocolitica*	Ampicillin	0 (0.0)	0 (0.0)	5 (100.0)
	Trimethoprim/sulfamethoxazole	2 (40.0)	2 (40.0)	1 (20.0)
	Ceftriaxone	0 (0.0)	4 (80.0)	1 (20.0)
	Streptomycin	0 (0.0)	0 (0.0)	5 (100.0)
	Amoxicillin/clavulanic acid	3 (60.0)	1 (20.0)	1 (20.0)
	Gentamycin	0 (0.0)	0 (0.0)	5 (100.0)
	Kanamycin	1 (20.0)	4 (80.0)	0 (0.0)
	Ciprofloxacin	5 (100.0)	0 (0.0)	0 (0.0)
	Chloramphenicol	0 (0.0)	1 (20.0)	4 (80.0)
	Erythromycin	0 (0.0)	4 (80.0)	1 (20.0)
	Nalidixic acid	3 (60.0)	2 (40.0)	0 (0.0)
	Tetracycline	1 (20.0)	1 (20.0)	3 (60.0)
Enterpathogenic bacteria	Antibiotic	Susceptibility pattern (%)		
		Sensitive	Intermediate	Resistant
*Aeromonas *species	Ampicillin	0 (0.0)	3 (75.0)	1 (25.0)
	Trimethoprim/sulfamethoxazole	2 (50.0)	1 (25.0)	0 (0.0)
	Ceftriaxone	4 (100.0)	0 (0.0)	0 (0.0)
	Streptomycin	2 (50.0)	2 (50.0)	0 (0.0)
	Amoxicillin/clavulanic acid	4 (100.0)	0 (0.0)	0 (0.0)
	Gentamycin	4 (100.0)	0 (0.0)	0 (0.0)
	Kanamycin	3 (50.0)	1(25.0)	0 (0.0)
	Ciprofloxacin	4 (100.0)	0 (0.0)	0 (0.0)
	Chloramphenicol	0 (0.0)	2 (50.0)	2 (50.0)
	Erythromycin	1 (25.0)	3 (75.0)	0 (0.0)
	Nalidixic acid	4 (100.0)	0 (0.0)	0 (0.0)
	Tetracycline	0 (0.0)	1 (25.0)	3 (75.0)
	Kanamycin	4 (19.0)	17 (81.0)	0 (0.0)
	Nalidixic acid	3 (60.0)	2 (40.0)	0 (0.0)
	Tetracycline	1 (20.0)	1 (20.0)	3 (60.0)
*Aeromonas *species	Ampicillin	0 (0.0)	3 (75.0)	1 (25.0)
	Trimethoprim/sulfamethoxazole	2 (50.0)	1 (25.0)	0 (0.0)
	Ceftriaxone	4 (100.0)	0 (0.0)	0 (0.0)
	Streptomycin	2 (50.0)	2 (50.0)	0 (0.0)
	Amoxicillin/clavulanic acid	4 (100.0)	0 (0.0)	0 (0.0)
	Gentamycin	4 (100.0)	0 (0.0)	0 (0.0)
	Kanamycin	3 (50.0)	1(25.0)	0 (0.0)
	Ciprofloxacin	4 (100.0)	0 (0.0)	0 (0.0)
	Chloramphenicol	0 (0.0)	2 (50.0)	2 (50.0)
	Erythromycin	1 (25.0)	3 (75.0)	0 (0.0)
	Nalidixic acid	4 (100.0)	0 (0.0)	0 (0.0)
	Tetracycline	0 (0.0)	1 (25.0)	3 (75.0)

 However, antimicrobial resistant to ampicillin, trimethoprim/sulfamethoxazole, ceftriaxone, streptomycin, amoxicillin/clavulanic acid, gentamycin, chloramphenicol, erythromycin and tetracycline was reported to be 5 (100.0%), 1 (20.0%), 1 (20.0%), 5 (100.0%), 1 (20.0%), 5 (100.0%), 4 (80.0%), 1 (20.0%), and 3 (60.0%), respectively, among *Yersinia* isolates. Even though none of *Aeromonas *isolates was resistant to trimethoprim/sulfamethoxazole, ceftriaxone, streptomycin, amoxicillin/clavulanic acid, gentamycin, kanamycin, ciprofloxacin, erythromycin and nalidixic acid, 1 (25.0%), 2 (50.0%) and 3 (75.0%) of *Aeromonas *isolates were resistant to ampicillin, chloramphenicol, and tetracycline, respectively.

## Discussion

Globally, enteric bacteria associated diarrhea continues to be a major cause of morbidity and mortality among children under 5 years (1). Enteric bacterial pathogens epidemiology shows variations between countries, and even between geographical regions within the same country (32). There has been a dramatic increase in the emergence of antimicrobial resistant enteric bacterial strains, which has made antibiotic choices for enteric infection treatment increasingly limited and more expensive (21). Thus, continuous epidemiological and antimicrobial resistance surveillances are fundamental in planning treatment.

Diarrheagenic *E. coli* predominates in this study followed by the rest namely *Shigella, Salmonella, Campylobacter, Yersinia *and* Aeromonus *species, in that order, highlighting the prominent role of *diarrhegenic E. coli* in enterobacteria associated diarrhea among children in Nairobi city, Kenya. These findings are consistent with previous studies involving diarrheic children in Kiambu (18) and Homa Bay counties, Kenya (19). As reviewed by (33), diarrhegenic* E. coli* persists longer in environmental reservoirs supporting the hypothesis of increased transmission of* E. coli* to human population (34), reinforcing the observation of this study. However, the findings of the present study are inconsistent with previous studies in the literature that demonstrated the dominancy of *Shigella *species, followed by diarrhegenic *E. coli, Salmonella *and* Yersinia *species among diarrheic children in Nairobi city, Kenya (3). This may be attributed to the fact that proved diarrhegenic *E. coli, *particularly EAEC, genome heterogeneity (35), using two virulence genes for EAEC detection decreased the rate of isolation in the previous study (3). Above all, this study detected higher prevalence of *Shigella* mono-infection (3.2%) than *Shigella/E. coli* co-infection (2.4%) suggesting that co-infections alter epidemiological dynamics of infectious disease due to pathogen synergism worsening disease severity and consequent mortality (36), and this observation is consistent with previous studies among diarrheic children in Kiambu county, Kenya (19), China (37), Zanzibar and Rwanda (38). Therefore, diarrhegenic *E. coli, Shigella, Salmonella, Campylobacter, Yersinia *and* Aeromonas *species are principal causes of childhood bacteria associated diarrhea justifying access to safe water, sanitation and hygiene among children in Nairobi city, Kenya. 

The current study observed high antimicrobial resistance rates indicating rapid and ongoing spread of antimicrobial-resistant organisms. Specifically, more than half of diarrheagenic *E. coli* isolates were resistant to ampicillin, trimethoprim/ sulfamethoxazole, streptomycin, kanamycin, chloramphenicol and tetracycline which is partly in agreement with studies in Nairobi city (17) and Meru county (14) but inconsistent with a previous study among the Maasai community of Narok and Kajiado counties that reported increased susceptibility to these antibiotics (39), possibly due to reduced antimicrobial use among the Maasai community who mostly practice traditional medicine (40). Over half of the *Shigella *isolates were resistant to streptomycin, chloramphenicol and tetracycline while resistance to ampicillin, ceftriaxone, amoxicillin-clavulanic acid, kanamycin, ciprofloxacin, and erythromycin occurred in more than half of *Salmonella* isolates, which partly agrees with a multisite study conducted in Kisii, Homa Bay and Migori counties, Kenya (22) but conflicts with a study in Zambia (41). Over half of *Campylobacter* isolates were resistant to ampicillin, chloramphenicol, and erythromycin which is consistent with the findings in Ethiopia (42) and disagrees with a study in China (43). At least half of *Yersinia* isolates were resistant to ampicillin, streptomycin, gentamycin and tetracycline which mirrors a study in Ethiopia (44) but disagrees with a study in Mexico (45). At least half of *Aeromonas *isolates were resistant to tetracycline and chloramphenicol which is in agreement with a study in Brazil (46) and disagrees with a study in China (43). These variations in resistance profiles between countries, within regions suggest significant differences in antibiotic use (47). Therefore, antimicrobial stewardship programs have to be developed to influence antibiotic use and prescribing behavior to ensure long-term availability of effective treatment for bacterial infections in Kenya.

This study had limitations. Other studies have detected viral, bacterial and parasitic gastroenteritis among Kenyan children (3, 4, 19) thus it is possible that the bacterial pathogens found may not be the sole cause of the diarrhea. Study participants were recruited within the hospital hence the prevalence of enteric bacteria and antimicrobial resistance does not represent community prevalence. We did not assay antimicrobial resistance genes. The presence of DAEC and AIEC pathotypes were not investigated in this study. We acknowledge the small sample size of *Aeromonas, Yersinia *and* Campylobacter *isolates assayed for antimicrobial resistance.

We conclude that diarrhegenic *E. coli, Shigella, Salmonella, Campylobacter, Yersinia *and* Aeromonas* species are important etiologies of diarrhea in children under five years of age, in Kenya. These pathogens are of public health importance since they are highly resistant to commonly prescribed antimicrobials*. *It is hoped the results of the present study will guide treatment of bacterial diarrheal diseases among children in the country.

## References

[1] (2018). Global Burden of Disease (2016) Diarrhoeal Diseases Collaborators, Estimates of the global, regional, and national morbidity, mortality, and aetiologies of diarrhoea in 195 countries: a systematic analysis for the Global Burden of Disease Study 2016. Lancet Infect Dis.

[2] The United Nations Children's Fund ( 2016). The State of the World's Children 2016. A fair chance for every child.

[3] Njuguna CI, Njeru E, Mgamb D, Langat A, Makokha D, Ongore (2016). Enteric pathogens and factors associated with acute bloody diarrhoea, Kenya. BMC Infect Dis.

[4] Swierczewski BE, Odundo MC, Koech JN, Ndonye RK, Kirera CP, Odhiambo (2013). Surveillance for enteric pathogens in a case-control study of acute diarrhea in Western Kenya. Trans R Soc Trop Med Hyg.

[5] Bundgaard-Nielsen C, Hagstrøm S, Sørensen S (2018). Interpersonal Variations in Gut Microbiota Profiles Supersedes the Effects of Differing Fecal Storage Conditions. Sci Rep.

[6] Gupta VK, Paul S, Dutta C (2017). Geography, Ethnicity or Subsistence-Specific Variations in Human Microbiome Composition and Diversity. Front Microbiol.

[7] Singh P, Teal TK, Marsh TL, Tiedje JM, Mosci R, Jernigan K (2015). Intestinal microbial communities associated with acute enteric infections and disease recovery. Microbiome.

[8] Mosites E, Sammons M, Otiang E, Eng A, Noecker C, Manor O (2017). Microbiome sharing between children, livestock and household surfaces in western Kenya. PLoS One.

[9] van Eijk AM, Brooks JT, Adcock PM, Garrett V, Eberhard M, Rosen DH (2010). Diarrhea in children less than two years of age with known HIV status in Kisumu, Kenya. Int J Infect Dis.

[10] Pavlinac PB, John-Stewart GC, Naulikha JM, Onchiri FM, Denno DM, Odundo EA (2014). High-risk enteric pathogens associated with HIV infection and HIV exposure in Kenyan children with acute diarrhoea. AIDS.

[11] Rinninella E, Raoul P, Cintoni M, Franceschi F, Miggiano GAD, Gasbarrini A (2019). What is the Healthy Gut Microbiota Composition? A Changing Ecosystem across Age, Environment, Diet, and Diseases. Microorganisms.

[12] Zilberman-Schapira G, Zmora N, Itav S, Bashiardes S, Elinav H, Elinav E (2016). The gut microbiome in human immunodeficiency virus infection. BMC Med.

[13] Boru WG, Kikuvi G, Omollo J, Abade A, Amwayi S, Ampofo W (2013). Aetiology and factors associated with bacterial diarrhoeal diseases amongst urban refugee children in Eastleigh, Kenya: A case control study. Afr J Lab Med.

[14] Karambu S, Matiru V, Kiptoo M, Oundo J (2013). Characterization and factors associated with diarrhoeal diseases caused by enteric bacterial pathogens among children aged five years and below attending Igembe District Hospital, Kenya. Pan Afr Med J.

[15] Brooks JT, Ochieng JB, Kumar L, Okoth G, Shapiro RL, Wells JG (2006). Surveillance for bacterial diarrhea and antimicrobial resistance in rural western Kenya, 1997-2003. Clin Infect Dis.

[16] Brooks JT, Shapiro RL, Kumar L, Wells JG, Phillips-Howard PA, Shi YP (2003). Epidemiology of sporadic bloody diarrhea in rural Western Kenya. Am J Trop Med Hyg.

[17] Sang WK, Oundo V, Schnabel D (2012). Prevalence and antibiotic resistance of bacterial pathogens isolated from childhood diarrhoea in four provinces of Kenya. J Infect Dev Ctries.

[18] Shah M, Kathiiko C, Wada A, Odoyo E, Bundi M, Miringu G (2016). Prevalence, seasonal variation, and antibiotic resistance pattern of enteric bacterial pathogens among hospitalized diarrheic children in suburban regions of central Kenya. Trop Med Health.

[19] Shah ME, Odoyo E, Wandera C, Kathiiko M, Bundi G, Miringu (2017). Burden of Rotavirus and Enteric Bacterial Pathogens among Children under 5 Years of Age Hospitalized with Diarrhea in Suburban and Rural Areas in Kenya. Jpn J Infect Dis.

[20] Swierczewski BE, Odundo EA, Koech MC, Ndonye JN, Kirera RK, Odhiambo CP (2013). Enteric pathogen surveillance in a case-control study of acute diarrhoea in the town of Kisii, Kenya. J Med Microbiol.

[21] Brogan DM, Mossialos E (2016). A critical analysis of the review on antimicrobial resistance report and the infectious disease financing facility. Global Health.

[22] Brander RL, Walson JL, John-Stewart GC, Naulikha JM, Ndonye J, Kipkemoi N (2017). Correlates of multi-drug non-susceptibility in enteric bacteria isolated from Kenyan children with acute diarrhea. PLoS Negl Trop Dis.

[23] Nyanga PL, Onyuka J, Webale MK, Were T, Budambula V (2017). Escherichia coli pathotypes and Shigella sero-groups in diarrheic children in Nairobi city, Kenya. Gastroenterol Hepatol Bed Bench,.

[24] Muloi D, Fevre EM, Bettridge J, Rono R, Ong'are D, Hassell JM (2019). A cross-sectional survey of practices and knowledge among antibiotic retailers in Nairobi, Kenya. J Glob Health.

[25] Rhee C, Aol G, Ouma A, Audi A, Muema S, Auko J (2019). Inappropriate use of antibiotics for childhood diarrhea case management -Kenya, 2009-2016. BMC Public Health.

[26] World Health Organization (2017). Antibacterial agents in clinical development – an analysis of the antibacterial clinical development pipeline. including tuberculosis.

[27] Cole ST (2014). Who will develop new antibacterial agents?. Philos Trans R Soc Lond B Biol Sci.

[28] World Health Organization The treatment of diarrhoea: A manual for physicians and other senior health workers.

[29] World Health Organization (2003). Manual for the laboratory identification and antimicrobial susceptibility testing of bacterial pathogens of public health concern in the developing world.

[30] Humphries RM, Ambler J, Mitchell SL, Castanheira M, Dingle T, Hindler JA (2018). CLSI Methods Development and Standardization Working Group Best Practices for Evaluation of Antimicrobial Susceptibility Tests. J Clin Microbiol.

[31] (2014). World Medical Association Declaration of Helsinki: ethical principles for medical research involving human subjects. J Am Coll Dent.

[32] Bublitz DC, PC Wright, JR Bodager, FT Rasambainarivo, JB Bliska, Gillespie TR (2014). Epidemiology of pathogenic enterobacteria in humans, livestock, and peridomestic rodents in rural Madagascar. PLoS One.

[33] Chekabab SM, Paquin-Veillette J, Dozois CM, Harel J (2013). The ecological habitat and transmission of Escherichia coli O157:H7. FEMS Microbiol Lett.

[34] Parvez SM, Azad R, Pickering AJ, Kwong LH, Arnold BF, Rahman MJ (2019). Microbiological contamination of young children's hands in rural Bangladesh: Associations with child age and observed hand cleanliness as proxy. PLoS One.

[35] Gomes TA, Elias WP, Scaletsky IC, Guth BE, Rodrigues JF, Piazza RM (2016). Diarrheagenic Escherichia coli. Braz J Microbiol.

[36] Olivo G, Lucas TM, Borges AS, Silva RO, Lobato FC, Siqueira AK (2016). Enteric Pathogens and Coinfections in Foals with and without Diarrhea. Biomed Res Int.

[37] Qu M, Lv B, Zhang X, Yan H, Huang Y, Qian H (2016). Prevalence and antibiotic resistance of bacterial pathogens isolated from childhood diarrhea in Beijing, China (2010-2014). Gut Pathog.

[38] Andersson M, Kabayiza JC, Elfving K, Nilsson S, Msellem MI, Martensson A (2018). Coinfection with Enteric Pathogens in East African Children with Acute Gastroenteritis-Associations and Interpretations. Am J Trop Med Hyg.

[39] Sang WK, Kariuki SM, Schnabel D, Boga HI, Waiyaki PG, Wamae CN (2011). Antibiotic susceptibility of Enteric pathogens from the Maasai community, Narok and Kajiado Districts, Kenya. Afr J Health Sci.

[40] Kimondo J, Miaron J, Mutai P, Njogu P (2015). Ethnobotanical survey of food and medicinal plants of the Ilkisonko Maasai community in Kenya. J Ethnopharmacol.

[41] Chiyangi H, Muma JB, Malama S, Manyahi J, Abade A, Kwenda G (2017). Identification and antimicrobial resistance patterns of bacterial enteropathogens from children aged 0-59 months at the University Teaching Hospital, Lusaka, Zambia: a prospective cross sectional study. BMC Infect Dis.

[42] Tafa B, Sewunet T, Tassew H, Asrat D (2014). Isolation and Antimicrobial Susceptibility Patterns of Campylobacter Species among Diarrheic Children at Jimma, Ethiopia. Int J Bacteriol,.

[43] Tian L, Zhu X, Chen Z, Liu W, Li S, Yu W (2016). Characteristics of bacterial pathogens associated with acute diarrhea in children under 5 years of age: a hospital-based cross-sectional study. BMC Infect Dis.

[44] Andualem B, Geyid A (2005). Antimicrobial responses of Yersinia enterocolitica isolates in comparison to other commonly encountered bacteria that causes diarrhoea. East Afr Med J.

[45] Novoa-Farias O, Frati-Munari AC, Peredo MA, Flores-Juarez S, Novoa-Garcia O, Galicia-Tapia J (2017). Susceptibility to rifaximin and other antimicrobials of bacteria isolated in patients with acute gastrointestinal infections in Southeast Mexico. Rev Gastroenterol Mex.

[46] Prediger KD, Pereira Rda S, Winckler Neto CH, Santos RC, Fadel-Picheth CM, Vizzotto BS (2012). A prospective study on Aeromonas in outpatients with diarrhea in the central region of Rio Grande do Sul State. Braz J Microbiol.

[47] Van Boeckel TP, Gandra S, Ashok A, Caudron Q, Grenfell BT, Levin SA (2014). Global antibiotic consumption 2000 to 2010: an analysis of national pharmaceutical sales data. Lancet Infect Dis.

